# Artificial intelligence in endodontics: A comprehensive review of applications and advancement

**DOI:** 10.6026/973206300223658

**Published:** 2026-06-30

**Authors:** Ameer Akhil Ahmed Shaik, Puspita Guha, Dhruvi Patel, Madhura Jadhav, Arindam Banik, Nirav Parmar, Mrunal Dave

**Affiliations:** 1 Department of Dental Surgery and General Dentistry, Narayana Dental College, Nellore, India; 2 Department of Dentistry, Agartala Government Dental College, Agartala, Tripura, India; 3 Department of Dental Surgery, College of Dental Sciences and Research Centre, Ahmedabad, Gujarat, India; 4 Department of Orthodontics and Dentofacial Orthopaedics, Bharati Vidyapeeth (Deemed to be University) Dental College and Hospital, Pune, Maharashtra, India; 5 Department of Conservative Dentistry and Endodontics, Guru Nanak Institute of Dental Sciences and Research, Kolkata, West Bengal, India; 6 Department of Conservative Dentistry and Endodontics, Faculty of Dental Science, Dharmsinh Desai University, Nadiad, Gujarat, India; 7 Department of Dentistry, Bethlehem Smile Design, Bethlehem, Pennsylvania, USA

**Keywords:** Artificial intelligence (AI), endodontics, application

## Abstract

The transformative impact of artificial intelligence (AI) on endodontics is gaining momentum. It highlights advancements in diagnostic accuracy, treatment planning and patient management. By integrating machine learning algorithms and imaging techniques, AI enhances clinical decision-making, reduces procedural errors and improves patient outcomes. Thus, data shows the current applications, challenges and future directions, proposing that AI will shape the future of endodontic practice and enhance the quality of dental care.

## Background:

Artificial Intelligence is revolutionizing health care by creating a bridge among technology and human interaction. Exhibiting intelligent behaviors to achieve specific objectives, the formal inception of AI took place in the 1950s and has since become a fundamental component of the healthcare landscape [1]. It holds the potential to significantly enhance diagnostic precision and clinical decision-making. Classical machine learning (ML) applications, especially in precision medicine, are gaining traction as they foresee treatment effectiveness by assessing on patient traits. Additionally, deep neural networks have transformed medical image analysis, showing improved results in tasks like cancer detection through lymph node evaluation. By implementing AI, healthcare can achieve greater diagnostic accuracy, reduces expanses and across all aspects of patient care [2, 3]. Robotic process automation, a subset of AI, simplifies organizational responsibilities and alleviates clinician fatigue by managing repetitive tasks. AI's ability to extract crucial medical insights improves decision-making in patient care, allowing for proactive strategies and tailored treatments. Additionally, machine learning algorithms enhance radiological evaluations, aiding in the early detection and diagnosis of diseases. The use of AI-driven technologies also digitizes interactions between providers and patients, enriching user experiences and personalizing healthcare services. AI has the potential to provide recent guidelines, researches and encourage innovation. However, misconceptions about AI can create inflated views and unrealistic hopes, highlighting the importance of accurate information and awareness in healthcare. The incorporation of AI marks a ground breaking chapter in healthcare, offering the potential for improved patient care, greater efficiency and significant advancements in the medical sector [2, 3]. While applications of AI in the dental industry are not yet commonplace, the advancements in technology have significantly influenced areas such as robotic assistance, dental imaging diagnostics, caries detection, radiography, pathology and electronic recordkeeping. As other dental specialties expand, research on AI in endodontics has also grown [4, 5]. Therefore, it is of interest to examine the diverse applications of artificial intelligence in endodontics and explore future prospects in the field.

## Artificial intelligence working model:

AI operates in two primary phases: training and testing. During training stage, the model learns from a set of training data, which includes previously collected examples such as patient records or diverse datasets pertinent to the task at hand. During this phase, the model identifies patterns and relationships within the data, adjusting its internal parameters to minimize errors in predictions. For instance, if the task involves diagnosing dental conditions, the training data may contain various cases of dental images along with their correct diagnoses. The model utilizes algorithms to analyze this data and optimize its parameters based on the outcomes [6, 7]. In the testing phase, the model's effectiveness is evaluated using a separate set of data known as the test set. This data differs from the training data and is used to evaluate the model's ability to apply its learned patterns to new, unseen instances. By applying the parameters established during training to the test set, the model generates predictions or classifications. The accuracy and reliability of these predictions are then measured, providing insights into the model's performance and utility in real-world applications. This two-phase approach ensures that AI systems can learn effectively and maintain high accuracy when applied to new scenarios [6, 7].

## Application of AI in endodontics:

Application of AI in endodontics is rapidly advancing, focusing on enhancing diagnostic accuracy, treatment planning and patient management. Here are some key areas where AI is being utilized in endodontics (Table 1).

## Endodontics diagnosis:

AI is transforming diagnostics in endodontics by enhancing accuracy and efficiency in identifying dental conditions. It analyses dental X-rays to detect root canal pathologies and lesions, automates caries detection to reduce human error and uses historical patient data for predictive diagnosis. Additionally, AI evaluates past cases to forecast treatment outcomes, employs deep learning for accurate image segmentation and integrates with electronic health records to provide diagnostic suggestions based on patient history. These advancements lead to more precise diagnoses, improved treatment planning and better patient outcomes in endodontics. In the field of endodontics, Aminoshariae *et al.* provided an extensive overview of AI models tailored for various tasks, such as studying root canal anatomy, detecting and diagnosing periapical lesions and root fractures and determining the working length for root canal treatment planning. They concluded that these AI models significantly assist clinicians in achieving precise diagnoses and treatment plans, ultimately enhancing treatment outcomes [5]. Building on this, Umer and Habib emphasized the use of AI in endodontics for diagnosis and treatment planning, reporting accuracy rates exceeding 90% for these tasks. Their findings further underscore AI's potential to elevate standards in endodontic practices [8]. Another study conducted by Tumbelaka *et al.* employed an artificial neural network (ANN) to differentiate among healthy pulp and diseased pulp using periapical radiographs. Their research, involving 20 teeth, demonstrated that digitizing direct reading radiography could improve the precision of pulpal diagnoses [9].

## Working length determination:

Accurate measurement of working length is essential for successful root canal treatments. One technique to evaluate working length is intraoral periapical (IOPA) radiography. Other methods include cone beam computed tomography (CBCT), electronic apex locators, digital tactile sensation and the patient's response to a paper or file point inserted into the root canal system. Clinically, radiography and electronic apex locators are the most commonly used techniques. The sharpness of digital radiography images is crucial for accurately evaluating the structure of the root canal system; however, multiple factors can influence radiographic interpretations and result in misdiagnoses. As a result, computer-based solutions are essential for reliably obtaining precise working lengths [7, 10]. A study conducted by Saghiri *et al.* revealed that artificial neural networks can serve as an effective supplementary tool for pinpointing the apical foramen in radiographs. This capability significantly boosts the accuracy of assessing the working length through radiographic analysis [11].

## Endodontic Imaging:

Radiographs and cone-beam computed tomography (CBCT) images are vital for endodontic diagnosis and treatment planning. However, their interpretation can be subjective, relying heavily on the clinician's expertise. Artificial intelligence, particularly deep learning models (DLMs) such as convolutional neural networks (CNNs), has demonstrated significant success in improving the accuracy and objectivity of image analysis. These models can effectively detect and classify endodontic pathologies, minimizing diagnostic errors and enhancing clinical decision-making [12]. For instance, Duan *et al.* utilized U-Net for tooth and pulp segmentation in CBCT images, employing a Feature Pyramid Network to ensure precise delineation of these anatomical structures [13].

## Identification of calcified and missed canals:

Artificial intelligence is increasingly used to identify neglected and calcified canals, which are common causes of endodontic failure. AI models can analyze CBCT scans to detect these canals, offering significant advantages over conventional methods. Additionally, AI aids in the automatic segmentation of root canal morphology, enabling clinicians to better understand and analyze complex anatomical structures [12].

## Decision making in endodontics:

Endodontic therapy boasts an impressive success rate of 90%, with only a 10% failure rate. To tackle this 10% challenge, theincorporation of AI technology presents a promising opportunity for endodontologists, as it aids in analyzing cases to determine whether retreatment or extraction is the best option. Researchers like Campo *et al.* have made strides in this field by developing a decision support system based on case-based reasoning. This system is expertly crafted to assess the potential success of retreatment, helping to minimize the number of unsuccessful procedures and accurately predicting outcomes. Remarkably, they achieved an accuracy of 84.4% in forecasting whether cases would conclude with additional treatment or extraction [14].

## Patient management and workflow optimization:

AI in endodontics significantly enhances patient management and workflow optimization. By automating administrative tasks such as appointment scheduling, billing and electronic health record management, AI reduces the administrative burden on dental practices. It also improves patient engagement through automated reminders and personalized communication. Furthermore, AI can analyze patient data to identify trends and monitor treatment progress, allowing for timely interventions. By streamlining processes and enhancing efficiency, AI helps clinicians focus more on patient care, ultimately leading to better patient outcomes and improved overall practice efficiency in endodontics [12].

## AI, Robotics and endodontics:

Endodontic robots, like DentiBot and visual-guided robotic therapeutic systems, are revolutionizing root canal procedures by automating tasks such as access preparation, cleaning, shaping and obturation. These systems assist with probing, drilling, cleaning and filling root canals while navigating complex anatomical structures. Equipped with force and torque sensors and patient tracking modules, they enable computer-aided treatment planning and control. This technology offers high precision in endodontic tasks and allows for real-time monitoring and adjustments during procedures. While providing enhanced accuracy and consistency, they also present challenges, including technical complexity, high costs and the need for extensive clinical validation and trials [15].

## Benefits of AI in endodontics:

AI in endodontics offers numerous advantages, including enhanced diagnostic accuracy through improved image analysis, which reduces the likelihood of misdiagnoses. AI-powered tools can assist in predicting treatment outcomes, optimizing case management and facilitating personalized treatment plans based on patient data. Additionally, the automation of administrative tasks streamlines workflows, allowing clinicians to focus more on patient care. AI-driven robotic systems improve precision in procedures, resulting in higher success rates and fewer complications. Overall, AI fosters improved efficiency, accuracy and patient satisfaction in endodontic practices [16, 17, 18].

## Challenges and limitations of AI in endodontics:

Despite its potential, AI in endodontics faces several challenges and limitations. The high complexity of technical implementation and the cost of advanced AI systems can be significant barriers for many practices. Additionally, the integration of AI into existing workflows may encounter resistance from clinicians who are accustomed to traditional methods. There are also concerns regarding the reliability of AI predictions and the need for extensive clinical validation and trials. Furthermore, data privacy and security issues related to patient information are critical considerations that must be addressed for widespread adoption of AI technologies in endodontics [19, 20, 21].

## Future Prospects of AI in endodontics:

The future of AI in endodontics appears promising, with ongoing advancements expected to further enhance diagnostic and treatment capabilities. As the integration of AI technologies continues, we can anticipate improved machine learning algorithms that will facilitate more accurate detection of complex canal systems and pathologies. Enhanced collaboration between AI systems and clinical practitioners will likely lead to more personalized treatment plans and better patient outcomes. Additionally, the development of cost-effective and user- friendly AI tools will make them accessible to a broader range of dental practices. Future research will focus on addressing current limitations, such as data privacy concerns and the need for clinical validation, ensuring that AI tools are not only effective but also safe and reliable [22, 23, 24].

## Conclusion:

The integration of artificial intelligence in endodontics holds significant promise for revolutionizing diagnosis, treatment planning and patient management. With its ability to enhance accuracy, streamline workflows and improve clinical decision-making, AI is poised to play a crucial role in the future of endodontic practice. However, challenges such as technical complexity, high costs and the need for validation must be addressed for successful adoption.

## Figures and Tables

**Table 1:** Summary of Applications of AI in Endodontics

**Application**	**Description**	**Benefits**
Diagnosis	AI algorithms analyze radiographs to identify endodontic issues	Improved accuracy, earlier detection of conditions
Treatment Planning	AI systems assist in creating personalized treatment plans	Increased efficiency, tailored approaches for patients
Predictive Analytics	AI predicts treatment outcomes based on historical data	Better patient management, informed decision-making
Workflow Optimization	AI streamlines scheduling and patient management processes	Reduced wait times, enhanced practice efficiency
Image Analysis	AI enhances image quality and features for better analysis	More reliable diagnosis, enhanced treatment planning
Patient Education	AI-driven tools provide educational resources for patients	Improved patient understanding and compliance
Post-Treatment Monitoring	AI monitors healing and success rates after procedures	Timely intervention, ongoing patient care
